# Long-Range Surface Plasmon-Polariton Waveguide Biosensors for Human Cardiac Troponin I Detection

**DOI:** 10.3390/s19030631

**Published:** 2019-02-02

**Authors:** Oleksiy Krupin, Pierre Berini

**Affiliations:** 1 School of Electrical Engineering and Computer Science, University of Ottawa, 800 King Edward Ave., Ottawa, ON K1N 6N5, Canada; a_krupin@hotmail.com; 2 Centre for Research in Photonics at the University of Ottawa, University of Ottawa, 25 Templeton, Ottawa, ON K1N 6N5, Canada; 3 Department of Physics, University of Ottawa, 150 Louis Pasteur, Ottawa, ON K1N 6N5, Canada

**Keywords:** optical biosensor, long-range surface plasmon-polariton, waveguide, fluoropolymer, human cardiac troponin I

## Abstract

Straight long-range surface plasmon-polariton (LRSPP) waveguides as biosensors for label-free detection are discussed. The sensors consist of 5-μm-wide 35-nm-thick gold stripes embedded in a low-index optical-grade fluoropolymer (CYTOP^TM^) with fluidic channels etched to the Au surface of the stripes. This work demonstrates the application of the LRSPP biosensors for the detection of human cardiac troponin I (cTnI) protein. cTnI is a biological marker for acute myocardial infarction (AMI), often referred to as a heart attack, which can be diagnosed by elevated levels of cTnI in patient blood. Direct and sandwich assays were developed and demonstrated over the concentration range from 1 to 1000 ng/mL, yielding detection limits of 430 pg/mL for the direct assay and 28 pg/mL for the sandwich assay (1 standard deviation), the latter being physiologically relevant to the early detection or onset of AMI. In addition, a novel approach for data analysis is proposed, where the analyte response is normalized to the response of the antibody layer.

## 1. Introduction

Biodetection using evanescent optical waveguide biosensors does not require molecular labeling (e.g., fluorescent or enzymatic tags) because the inherent principle of detection is based on sensing changes in refractive index at the sensor surface as biomolecules (mass) bind thereon. This allows for direct real-time detection, which in addition, yields kinetic parameters for the bio-interaction [1]. Other advantages such as nonlaborious sample preparation, no requirements for well-trained personnel, and reduced consumption of reagents make optical biosensors competitive against lab-based diagnostic techniques such as ELISA (enzyme-linked immunosorbent assay), SDS-PAGE (sodium dodecyl sulfate polyacrylamide gel electrophoresis), or bacterial culture. Currently, the field of optical biosensors makes extensive use of surface plasmon resonance (SPR) phenomena in the Kretschmann–Raether prism-coupled configuration [2,3]. SPR biosensors comprise a significant share of the total label-free biosensor market [4].

Long-range surface plasmon-polaritons (LRSPPs) are surface plasmon waves that can propagate over appreciable lengths along a metal stripe bounded by dielectrics of similar refractive index [5]. Konopsky and Alieva [6] reported the excitation of LRSPPs on a thin 5 nm Au film bounded by air on one side and on the other by a 1D photonic crystal constructed as a Ta_2_O_5_/SiO_2_ multilayer stack. The LRSPPs can be excited optically in various arrangements including end-fire (butt-coupling). The propagation length of LRSPPs can extend to centimeters, whereas that of conventional SPPs (as used in SPR biosensors) is limited to ~80 μm, which makes the former favorable for biosensing due to the significantly increased optical interaction length with the sensing medium. The penetration depth of LRSPPs into the sensing medium is also larger, ~2000 nm compared to ~200 nm for conventional SPPs, which opens new sensing applications such as monitoring processes within biological cells, or the use of a thick sensing layer constructed with, e.g., a hydrogel dextran matrix, to enable analyte capture along the 3rd dimension. Optimal conditions for LRSPP propagation along metal stripes require that the top and bottom bounding dielectrics have similar refractive indices (RIs). Biologically-compatible solutions have a RI close to that of water (n ~ 1.32) so the bottom cladding may be comprised of a low-RI fluoropolymer material such as CYTOP^TM^ (Asahi) or Teflon (Dupont).

Only a few studies exploiting LRSPPs in SPR prism-based sensor geometries via the inclusion of a Teflon layer have been reported. The aforementioned system produced enhanced sensing capabilities for bulk RI sensing [7] and *E. coli* detection [8]. In addition, the larger LRSPP field penetration depth was utilized for studying the effects of toxins on cancer cells [9] and monitoring cellular micromotion within fibroblast cells [10]. Compared to modified SPR prism-based sensors, LRSPP waveguides have an additional advantage: due to wave confinement in the plane transverse to the direction of propagation, various integrated waveguide configurations such as Y-junctions, S-bends, and phase-sensitive Mach-Zehnder Interferometers (MZIs) can be constructed [11,12]. For instance, Au waveguide MZIs have been demonstrated for bulk sensing, enabling a detection limit about one order of magnitude better than straight LRSPP waveguides [13]. 

Previously, LRSPP waveguide biosensors have been successfully tested and demonstrated for a broad range of biodetection problems, such as the detection of A-positive human blood type based on ABO blood grouping [14] and the detection of Dengue infection based on the presence of dengue-specific IgM [15] and nonstructural protein 1 (NS1) in patient plasma [16]. B-cell leukemia in patient serum was detected by comparing the relative ratio of human IgG kappa (IgGκ) to human IgG lambda (IgGλ) in each patient sample [17]. Urinary tract infection (UTI) detection was performed on anti-Gram-positive and anti-Gram-negative antibody-functionalized Au surfaces using either *E. coli* or *S. epi* bacteria in urine [18]. In addition, the viability of bacteria was assessed by correlation to output power fluctuations (noise), which was observed to be high only when live bacteria was present on the surface [19], most likely due to bacterial micromotion (quiver). The sensitivity of LRSPP biosensors to small molecules was also demonstrated by detecting a monolayer of N-hydroxysulfosuccinimide (NHS, ~200 Da) on a carboxyl-terminated Au sensor surface [20].

Acute myocardial infarction (AMI), also known as a heart attack, is a type of heart disease and is the leading cause of death amongst all types of heart ailments [21]. AMI is caused by a blockage of blood flow, which results in damage to the heart muscle and the release of some proteins (biomarkers) into the blood stream. One of the most important biomarkers for AMI diagnostics is human cardiac troponin I (cTnI). The investigation of cTnI as a biomarker started in the 1990s. Currently, cTnI detection along with electrocardiograms (ECGs) are routine diagnostic approaches for AMI. The cut-off value for ruling-in or ruling-out AMI based on the cTnI concentration in serum has decreased significantly over the past decades, from 3.1 ng/mL in the 1990s [22] down to 26.7 pg/mL currently [23], possibly due to improvements in detection methods. After the onset of AMI, cTnI blood levels continue to rise for 12 to 24 h, reaching up to 1000 ng/mL and remaining highly elevated for several days [24]. Upon admission to the hospital, a suspected AMI patient is monitored by measuring cTnI levels every three hours to assess the treatment and progression of the condition [25]. Although a number of cTnI sensing platforms are emerging and obtaining FDA clearance [26], many hospitals still rely on a standard cTnI ELISA test, which does provide a high sensitivity (LOD ~ 5 pg/mL, [27]), but is time-consuming, requires a large amount of (bio)reagents, and the involvement of well-trained personnel. Optical biosensors have been used for cTnI detection but no compact commercial systems are available. However, commercial prism-based SPR systems have been utilized to detect cTnI to a LOD of 250 pg/mL using a sandwich assay [28] and to 68 pg/mL using a direct assay [29]. Thus, plasmonic biosensors have potential as cTnI sensors for monitoring and detection due to their high sensitivity and simple bioassays. 

We briefly describe in the following sections the structure and assembly of our LRSPP biosensor, and present results on the detection of human cardiac troponin I. 

## 2. Overview of LRSPP Waveguide Biosensors

### 2.1. LRSPP Biosensor Structure and Fluidic Assembly

**Figure 1:**
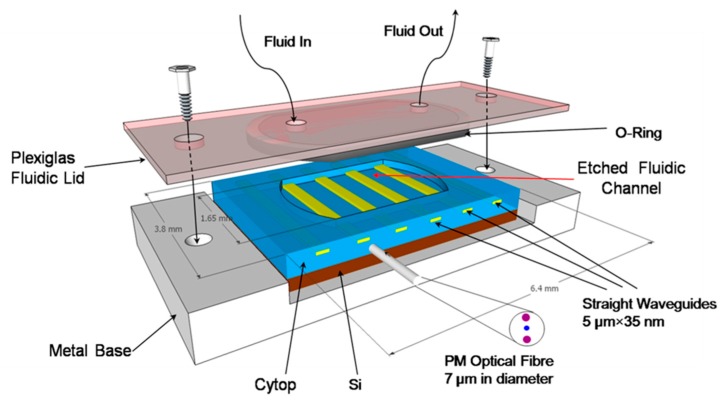
Schematic representation of the sensor on a metal base with a fluidic lid; the volume of the fluidic cell is 20 µL.

LRSPP biosensing chips (3.2 × 6.4 mm^2^) are fabricated on a 100 mm Si wafer comprising ~300 die [30]. Each device consists of a series of straight Au stripes (5 μm wide, 35 nm thick) embedded in CYTOP as sketched in Figure 1, which enables multiplexed sensing (in principle). A fluidic cavity is fabricated by etching the top CYTOP cladding down to the Au surface, thus exposing sections of the Au stripes over a length of 1.65 mm for biosensing—each individual stripe is a biosensor. A chip is integrated with external fluidic elements by placing it onto a metal base and securing it to a custom Plexiglass lid using screws. The lid comprises a fluorocarbon O-ring to provide a fluidic seal and two holes within the O-ring region with attached Pico tubing (550 μm outer diameter, 250 μm inner diameter) for fluid access. The volume of the fluidic cell is 20 µL. The fluids are supplied by a syringe pump. LRSPPs are excited by butt-coupling a polarization-maintaining single-mode fiber (PM-SMF), 7 μm in core diameter, to the input facet. In order to maintain optical symmetry near the waveguide (n_CYTOP_ = 1.335), a standard phosphate buffered saline (PBS, pH = 7.4) is doped with glycerol (16.5% w:w, PBS/Gly) to reach a slightly higher RI of 1.338, which has been found to provide very good sensitivity in LRSPP waveguide biosensors [31].

### 2.2. Optical Interrogation

**Figure 2:**
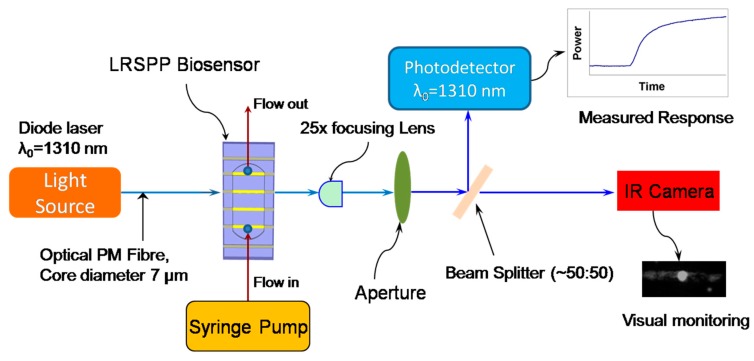
Schematic of the optical setup for biosensing using a straight long-range surface plasmon-polariton (LRSPP) sensor.

The optical interrogation setup (Figure 2) includes the light source (diode laser emitting at λ_0_ = 1310 nm), connected to the PM-SMF. Fiber alignment to the waveguide is performed by manipulating two positioning stages: a 3-axis stage supporting a biosensor with fluidics and a second 6-axis stage for the input PM fiber. The syringe pump is incorporated alongside to provide fluids to the system. A 25× objective lens is firmly affixed to the optical table and used to define the optical axis of the set-up and approximately collimate the output beam. Background radiation in the exiting beam is minimized using a pinhole aperture before it is sent to the 50:50 beam splitter. One part of the split beam is sent to the infrared camera for visual monitoring and alignment, and the other part is sent to a power meter to record real-time power changes during an experiment. Labview was used to control the setup and perform data acquisition.

## 3. Detection of Human Cardiac Troponin I (cTnI)

Protein G (PG) is a streptococcal protein that has a strong affinity to the crystallizable fragment (Fc) of IgGs, and thus is capable of orienting the IgG molecules in an “upward” direction, exposing the antigen-binding fragments (Fabs) to the solution carrying analyte. As a result, the immunosurface has a higher avidity compared to the alternative (and more common) functionalization approach of using a thiol-based self-assembled monolayer (SAM) with carbodiimide chemistry [32] to covalently attach IgG in a nonspecific orientation. Although some PG functionalization strategies using modified Protein G have been proposed [33,34], the simple adsorption of PG directly onto bare Au at a pH of 7.4 and room temperature works well [35].

Here we demonstrate direct and sandwich immunoassays for cTnI detection in PBS buffer, using Protein G as the linking chemistry to functionalize the surface of LRSPP waveguide biosensors. Our objectives are to demonstrate these cTnI assay formats on LRSPP biosensors, determine the limit of detection for cTnI in clean fluids, and investigate the range of concentrations over which cTnI does not precipitate onto a biosensor surface (precipitation is suspected to occur with this protein which can preclude quantitative biodetection results). 

### 3.1. Materials

2-Isopropanol (IPA,733458), acetone (270725), glycerol (49767), lyophilized bovine serum albumin (A0281), heptane (34873), Protein G (P4689), sodium dodecyl sulfate (71725), and phosphate buffered saline (PBS, P5368) 0.01 M, pH 7.4 were obtained from Sigma-Aldrich. PBS solution was prepared by dissolving packaged salts in 1 L of distilled/deionized (DDI H_2_O) water. Natural Cardiac Troponin I protein was purchased from Abcam (ab9936). Goat polyclonal anti-human Troponin I IgGs were purchased from Fitzgerald Industries International (70-B9085GA01-A0).

### 3.2. Surface Functionalization and Bioassay

**Figure 3:**
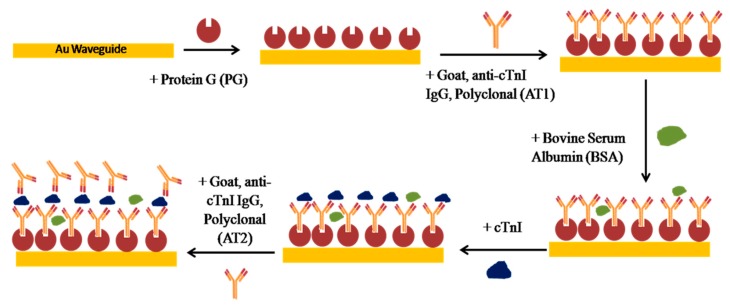
Functionalization strategy and sandwich bioassay developed for the detection of cTnI.

Once a biosensor chip was incorporated into the setup, the system was allowed to stabilize while flowing PBS/Gly buffer at a 20 μL/min flow rate. Surface functionalization was performed by first injecting 50 μg/mL of Protein G in PBS/Gly (PG) at 20 μL/min over a cleaned bare Au waveguide for 15 min. This was followed by flowing polyclonal goat anti-human cTnI IgG (AT1, 200 μg/mL in PBS/Gly) at 5 μL/min for 90 min. Nonspecific binding sites were blocked by injecting bovine serum albumin (BSA, 1 mg/mL in PBS/Gly) at 20 μL/min for 10 min. The bioassay was carried out by injection of cTnI in PBS/Gly for 30 min for the direct detection, followed by the injection of goat anti-human cTnI IgG (AT2, 200 μg/mL in PBS/Gly) for another 30 min to complete the sandwich assay. Figure 3 gives a sketch of the functionalization approach and the bioassay developed. 

Several devices were used to perform seven experimental runs with solutions of cTnI in PBS/Gly buffer (n = 1.338) of concentration 1, 10, 100, 1000, 10,000, and 20,000 ng/mL. After each experiment the whole system was washed with 5% (w:w) sodium dodecyl sulfate (SDS) in DDI H_2_O for 30 min, followed by pure DDI H_2_O for 15 min. The fluidic system was disassembled and any remaining organic constituents on the device were removed by exposure in a UV/Ozone chamber for 30 min (15 min lamp-on followed by 15 min lamp-off). This device cleaning procedure provides excellent surface regeneration and allows for a single device to be reused 4 to 6 times [14,17,36]. Nonetheless, more than one device was used throughout the whole experimental set. In order to resolve device-to-device variation issues, normalization of the data was performed as briefly described below, following [17].

### 3.3. Results and Discussion

#### 3.3.1. Protein G/IgG Interaction

**Figure 4:**
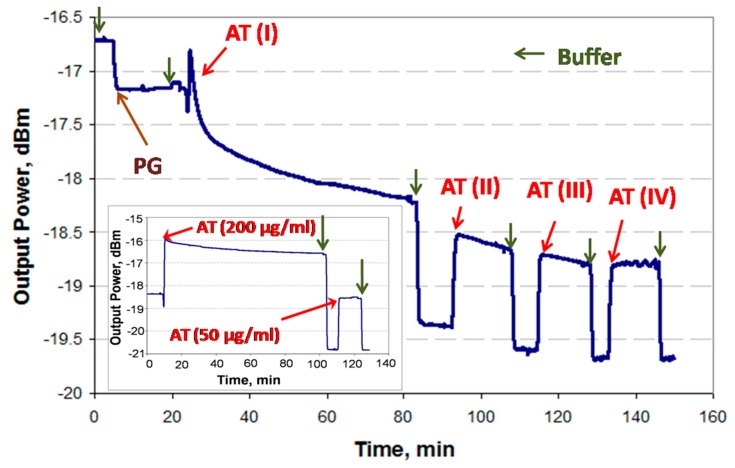
Real-time interaction of Protein G and goat polyclonal anti-human cTnI IgG (AT) as revealed by an LRSPP biosensor. The PG/IgG functionalized surface was tested by the periodic injection of AT (200 μg/mL) until the difference between baselines disappeared. The inset shows a different experimental run where the first injection of AT was carried out for 90 min straight.

Incomplete binding to the PG-coated surface during the first injection of IgGs (i.e., AT1, Figure 4) can introduce significant errors in a sandwich assay because the second injection of IgGs (i.e., AT2, Figure 4) may lead to binding with unoccupied Protein G sites, producing a false positive. In order to avoid this problem, a thorough investigation of the PG/IgG interaction was performed.

It has been experimentally observed in the case of high concentrations of IgG (>50 μg/mL) that the binding response of IgG to the PG surface does not always fully saturate. This can be attributed to IgG forming a multilayer due to accumulation in stagnant regions of the fluidic channel (i.e., in dead volumes) near the optical path. Thus, it would be ineffective to wait for the response to stabilize in order to proceed to the next step (i.e., cTnI analyte injection). In order to resolve this issue and experimentally establish the time required to achieve full coverage of IgGs, the PG-functionalized surface was first exposed to goat anti-troponin IgG (AT, 200 μg/mL in PBS/Gly) for 60 min (saturation was not observed), followed by three additional wash and injection steps, as shown in Figure 4. (Bulk steps are observable at fluid exchange points due to differences in the refractive index of the solutions used.) 

One can identify full coverage of IgG by noting the absence of change in the baseline level after two sequential PBS/Gly wash steps, as observed after the fourth AT injection step (AT-IV). The total time required to achieve an unchanging baseline signal was 90 min. In order to confirm this result, an experiment was conducted by injecting 200 μg/mL of AT in PBS/Gly for the full 90 min after the initial PBS/Gly wash step, followed by injecting 50 μg/mL of AT in PBS/Gly for 15 min, as shown in the inset of Figure 4, revealing no baseline signal change. Thus, it was concluded that a 90 min injection of 200 μg/mL AT suffices to completely functionalize the surface.

#### 3.3.2. Full Experimental Response

**Figure 5:**
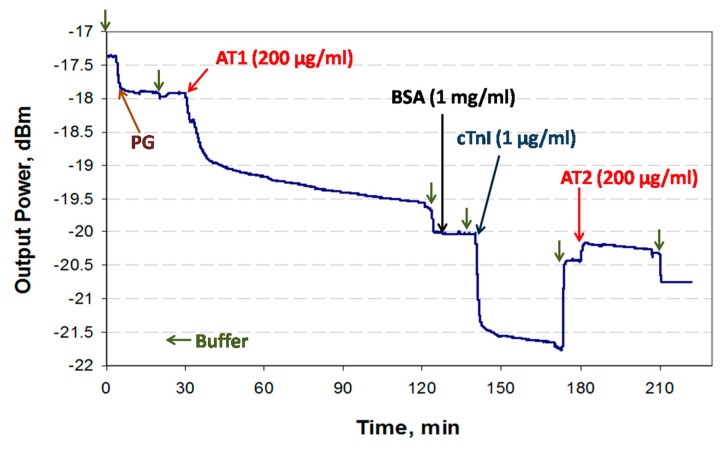
Example sensorgram of a sandwich bioassay for the detection of cTnI.

An example sensorgram of the sandwich assay developed for the detection of cTnI (1 μg/mL in PBS/Gly) is presented in Figure 5. 

Generally, it was observed that the relative signal changes due to full coverage of PG (~0.5 dB) and AT1 (~2.1 dB) correspond to their relative molecular weights (~65 kDa and ~150 kDa), which is consistent with the fact that the attenuation of the LRSPP depends on the adsorbed mass [31]. The bare Au surface has a strong affinity for proteins via chemi- and physisorption processes compared to immunoreactions, as observed in the binding responses; the binding response is much steeper for PG adsorption. Although saturation of the AT1 response is not observed after 90 min of injection, it was assumed that the surface was fully covered and the remaining decrease in signal was due to other artefacts (see Section 3.3.1). After AT1 functionalization, the surface was washed with PBS/Gly and blocked with 1 mg/mL of BSA in PBS/Gly. There is no apparent change in signal during the BSA injection or when comparing the PBS/Gly baseline levels before and after the injection, which suggests that the waveguide surface was fully covered following injection with AT1. At ~140 min (baseline level of −20.05 dBm), the cTnI solution was injected, and after the initial bulk step, a binding curve observed due to cTnI binding with AT1. After a 30 min flow at a rate of 5 μL/min, the fluidic cell was washed with PBS/Gly leading to a new baseline level of −20.5 dBm (following the bulk step). This series of steps completes a direct assay cycle. For the sandwich bioassay, goat anti-human cTnI IgG (AT2) was injected for 30 min at a 5 μL/min flow rate, then the fluidic cell was washed with PBS/Gly. After the bulk step, binding of AT2 to cTnI can be clearly observed, as well as the difference in baseline levels before and after AT2 injection. The baseline levels (after the PBS/Gly washes) were used in the data analysis (Section 3.3.3).

#### 3.3.3. Data Analysis

The relationship between the change in surface mass density and the output power due to adlayer formation is expressed as [31

$$\Delta \Gamma =\frac{1}{k_{2}}\frac{\left(n_{a}-n_{c}\right)}{\delta n/\delta c}\left(\frac{P_{out}\left(a_{1}\right)}{P_{out}\left(a_{0}\right)}-1\right)$$

where ΔΓ is the change in surface mass density (g/m^2^), *n_a_* and *n_c_* are the refractive index of the adlayer material and the sensing fluid, respectively, *k*_2_ is a constant that is specific for an individual sensor (varies with fabrication), ∂*n*/∂*c* is the partial change in refractive index of a solution relative to the analyte concentration (adlayer material), and *P_out_*(*a*_0_) and *P_out_*(*a*_1_) are the output powers (in W) before and after adlayer formation, respectively.

Due to imperfections during fabrication, devices can differ slightly in terms of performance. In order to eliminate this factor, the cTnI and AT2 responses were normalized to the AT1 response:

$$\frac{\Delta \Gamma \left(analyte\right)}{\Delta \Gamma \left(AT1\right)}=\frac{{\left(\frac{P_{out}\left(a_{1}\right)}{P_{out}\left(a_{0}\right)}-1\right)}_{analyte}}{{\left(\frac{P_{out}\left(a_{1}\right)}{P_{out}\left(a_{0}\right)}-1\right)}_{AT1}}$$

where *analyte* refers to cTnI or cTnI + AT2 for the direct and sandwich assays, respectively. The term involving *k*_2_ cancels out because it remains constant throughout an experiment. Thus, device-to-device variations are removed and the normalized responses can be compared between all experimental runs. 

**Figure 6:**
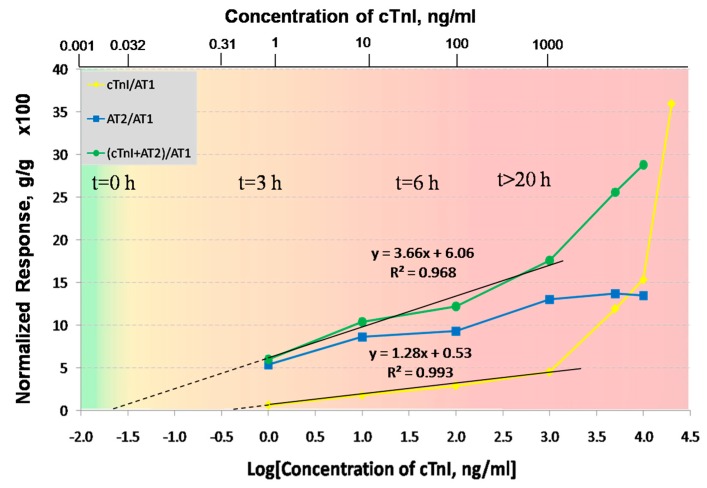
Summary plot of the normalized LRSPP biosensor responses over the cTnI concentration range of 1 to 20,000 ng/mL using direct and sandwich bioassays. All the responses were normalized with respect to the surface mass load of the first adlayer of goat anti-human cTnI IgGs (AT1), and multiplied by a factor of 100 for clarity. The error bars are significantly smaller than the response values, therefore are not visible in the plot. The superimposed color gradient and timescale show how the concentration of cTnI in blood increases as an AMI progresses in time [28].

Figure 6 summarizes the responses of the LRSPP biosensor for the seven concentrations of cTnI used in the experiments. The yellow line corresponds to the normalized response of the direct assay (ΔΓ[cTnI]/ΔΓ[AT1]), the blue line to the normalized response of the second antibody (ΔΓ[AT2]/ΔΓ[AT1]), and the green line to the normalized response of the sandwich assay (ΔΓ[cTnI + AT2]/ΔΓ[AT1]). The normalized responses were multiplied by a factor of 100 for easier visualization. The direct assay produces a linear-log relationship for cTnI concentrations of 1 to 1000 ng/mL. However, above 1 μg/mL the response becomes nonlinear. This is likely due to cTnI precipitation, which is thought to happen at concentrations above 1 μg/mL in the absence of carrier proteins (as mentioned by the cTnI supplier [37]). Furthermore, the response of AT2 saturates at 1 mg/mL, which confirms that the increase in the cTnI response is not an experimental artifact but probable cTnI precipitation. The sandwich assay above 1 μg/mL also produces a significantly higher slope than below, also due to cTnI precipitation. Thus the concentration range over which cTnI does not precipitate limited to a maximum of 1000 ng/mL. The normalized responses over the meaningful cTnI concentration range of 1 to 1000 ng/mL were fitted to straight lines, with R^2^ goodness of fit values of 0.993 for the direct assay and 0.968 for the sandwich assay. In order to determine the limit of detection (LOD) for both bioassays, the straight line fits were extrapolated to zero, yielding LODs of 386 and 22 pg/mL for the direct and sandwich assays, respectively. However, this approach does not take into account the system noise, so to obtain more realistic LOD estimates, the standard deviation *δ* over time of each baseline response was computed over ~5 min (noise) and incorporated into the ΔΓ/ΔΓ calculations for each data point as described in the Supplementary Material. The worst case standard deviation for the direct assay was *δ* = 0.061 (g/g × 100), which makes the LOD 430 pg/mL (Table 1). For the sandwich assay the worst case standard deviation was found to be *δ* = 0.404 (g/g × 100), which makes the LOD 28 pg/mL. (The noise is significantly smaller than the normalized responses and so cannot be observed in Figure 6.) The LODs including noise were estimated for both assays by equating the ordinate of the linear model to the corresponding *δ* and extracting the abscissa (Log([cTnI])) as the LOD (1 × standard deviation was used).

A gradient color scale was added to Figure 6 to illustrate how the concentration of cTnI in blood changes as a myocardial infarction progresses in time [24]. Ignoring our extrapolated LODs, the LRSPP biosensor can clearly differentiate between a healthy patient and a patient suffering from AMI after ~3 h of infarction onset (1 ng/mL is the lowest concentration that we have tested), using either the direct or sandwich assays. In addition, provided that the sensor surface is prefunctionalized with AT, the detection times are ~40 min for the direct assay and ~90 min for the sandwich assay (unoptimized), which makes it possible to monitor an infarction patient as suggested by a current clinical protocol [25]. Although not tested below 1 ng/mL, our LRSPP sensor with a sandwich assay has the potential to detect earlier stages (<3 h) or even the onset of AMI, given our extrapolated LOD of 28 pg/mL, which is only slightly higher than the threshold established by the medical community of 26.7 pg/mL. 

## 4. Conclusions

LRSPP waveguide biosensors are fabricated using common semiconductor lithographic techniques at a wafer scale, so the overall cost of this novel plasmonic platform can be significantly reduced compared to conventional SPR technology. This sensor can be easily modified with proper (bio)chemistry for a broad scope of detection problems which are based on affinity binding. Multiple waveguides integrated on a single chip can add multiplexing and expand the platform’s capabilities. Furthermore, a single chip can be reused several times via a simple process of UV/ozone treatment which can potentially reduce the cost. 

LRSPP biosensor was also demonstrated for the label-free detection of cTnI—a myocardial infarction biomarker—using direct and sandwich bioassays. The sensor surface was functionalized with goat anti-human cTnI IgG via Protein G/IgG interaction. The sandwich assay implemented on this biosensor exhibits strong potential for cTnI detection, producing an (extrapolated) limit of detection of 28 pg/mL. The extrapolated limit of detection of the direct assay is 430 pg/mL. Both values are in the range of the physiologically relevant cTnI concentrations for the purposes of diagnosis and monitoring of AMI. A benefit of these rather simple bioassays is the short assay time of 40 to 90 min, which is expected to decrease with optimization of the biosensor and the bioassay protocol. 

Overall, the presented LRSPP biosensor can potentially provide a low-cost and rapid solution to various detection problems that exist in fields such as healthcare, pharmacology, environmental monitoring, or food safety. 

## Figures and Tables

**Table 1:** Noise (standard deviation of the baseline, *δ*) over the cTnI concentration range of 1 to 1000 ng/mL for the direct and sandwich assays, computed using Equations (12S) and (19S) (Supplementary Materials). The worst cases are bolded.

Concentration of cTnI, ng/mL	*δ* (Direct Assay, g/g × 100)	*δ* (Sandwich Assay, g/g × 100)
1	0.043	0.315
10	0.055	0.337
100	0.030	0.297
1000	**0.061**	**0.404**

## Supplementary Materials

The following are available online at http://www.mdpi.com/1424-8220/19/3/631/s1.
