# Supplementary material for: Long-Range Surface Plasmon-Polariton Waveguide Biosensors for Human Cardiac Troponin I Detection
Source: Sensors (Basel). 2019 Feb 2;19(3):631. doi: 10.3390/s19030631 (PMC6386922; doi:10.3390/s19030631)
Supplement: Supplementary file 1 [file sensors-19-00631-s001.pdf]

# Long-Range Surface Plasmon-Polariton Waveguide Biosensors for Human Cardiac Troponin I Detection

**Oleksiy Krupin[1,2] and Pierre Berini[1,2,3,*]**

[1] School of Electrical Engineering and Computer Science, University of Ottawa, 800 King Edward Ave., Ottawa K1N6N5, Canada; a_krupin@hotmail.com
[2] Centre for Research in Photonics at the University of Ottawa, University of Ottawa, 25 Templeton, Ottawa, Ontario K1N 6N5, Canada
[3] Department of Physics, University of Ottawa, 150 Louis Pasteur, Ottawa K1N 6N5, Canada;
[*] Correspondence: berini@eecs.uottawa.ca; Tel: +001 613 562-5800 x6316

Received: 28 December 2018; Accepted: 29 January 2019; Published: date

## S1. Means and Variances

Six basic equations for variance estimation are used throughout our analysis:

To find the variance of the ratio of two random variables, Var(X/Y), a Taylor expansion can be used:

$$Var\left[\frac{X}{Y}\right] \approx Var\left[\frac{1}{\mu_Y^2}Var\left[X\right]+\frac{\mu_X^2}{\mu_Y^4}Var\left[Y\right]-2\frac{\mu_X}{\mu_Y^3}Cov\left[X,Y\right]\right].$$

Here, $X=P_{out}(a_1)$ and $Y=P_{out}(a_0)$, and since they are independent variables, Cov(X,Y)=0, so the final expression for Var(X/Y) is:

$$Var\left[\frac{X}{Y}\right] \approx Var\left[\frac{1}{\mu_Y^2}Var\left[X\right]+\frac{\mu_X^2}{\mu_Y^4}Var\left[Y\right]\right]. \quad (1S)$$

The addition of a constant to a random variable does not change the variance:

$$Var\left[X+c\right]=Var\left[X\right]. \quad (2S)$$

The addition of a constant to the mean (μ) changes the mean by the same value:

$$\mu\left[X+c\right]=\mu\left[X\right]+c. \quad (3S)$$

If the variable is scaled by a constant, the variance is scaled by the square of that constant:

$$Var\left[aX\right]=a^2Var\left[X\right]. \quad (4S)$$

The variance of the summation of two random variables is given by:

$$Var\left[aX+bY\right]=a^2Var\left(X\right)+b^2Var\left(Y\right)+2abCov\left(X,Y\right).$$

Output powers are independent variables, so again Cov(X,Y)=0 and the above simplifies to:

$$Var\left[aX+bY\right]=a^2Var\left(X\right)+b^2Var\left(Y\right). \quad (5S)$$

Means (μ) of the responses were calculated by determining the time-average of a baseline response (prior to analyte injection) over 5 min, taking into account 600 data points. Variances (*Var*) of the corresponding responses were calculated by determining the variance of the response over the same timeframe as for the mean. Since the same number of datapoints (600) was used to calculate all the means at the baseline, the mean of the ratio is equal to the ratio of individual means:

$$\mu\left[\frac{X}{Y}\right]=\frac{\mu_X}{\mu_Y}. \quad (6S)$$

**S2. Variance of normalized direct assay responses**

The normalized response for cTnI/AT1 (g/g ×100) is expressed as:

$$\frac{\Delta\Gamma(cTnI)}{\Delta\Gamma(AT1)} = \left[\frac{\left(\frac{P_{out}(a_1)}{P_{out}(a_0)}-1\right)_{cTnI}}{\left(\frac{P_{out}(a_1)}{P_{out}(a_0)}-1\right)_{AT1}}\right]\times 100 \,. \quad (7S)$$

with the available data being:

| Means | Variances |
|---|---|
| $\mu_{cTnI(1)}$; $\mu_{cTnI(0)}$; | $V_{cTnI(1)}$; $V_{cTnI(0)}$; |
| $\mu_{AT1(1)}$; $\mu_{AT1(0)}$; | $V_{AT1(1)}$; $V_{AT1(0)}$; |

The variance of the numerator (cTnI), using Eqs.(1S) and (2S) is expressed as:

$$Var(cTnI) = Var\left(\frac{P_{out}(a_1)}{P_{out}(a_0)}-1\right)_{cTnI} = \frac{V_{cTnI(1)}}{\left(\mu_{cTnI(0)}\right)^2} + \frac{\left(\mu_{cTnI(1)}\right)^2 \times V_{cTnI(0)}}{\left(\mu_{cTnI(0)}\right)^4} \,. \quad (8S)$$

The variance of the denominator (AT1), using Eqs. (1S) and (2S):

$$Var(AT1) = Var\left(\frac{P_{out}(a_1)}{P_{out}(a_0)}-1\right)_{AT1} = \frac{V_{AT1(1)}}{\left(\mu_{AT1(0)}\right)^2} + \frac{\left(\mu_{AT1(1)}\right)^2 \times V_{AT1(0)}}{\left(\mu_{AT1(0)}\right)^4} \,. \quad (9S)$$

The variance of the ratio (cTnI/AT1), using (1S) and (6S) is given by:

$$Var\left(\frac{cTnI}{AT1}\right) = \frac{Var(cTnI)}{\left(\mu_{AT1(1)}/\mu_{AT1(0)}\right)^2} + \frac{\left(\mu_{cTnI(1)}/\mu_{cTnI(0)}\right)^2 \times Var(AT1)}{\left(\mu_{AT1(1)}/\mu_{AT1(0)}\right)^4} \,. \quad (10S)$$

Using Eq. (4S), the total variance is expressed as:

$$Var\left(\frac{cTnI}{AT1}\times 100\right) = Var\left(\frac{cTnI}{AT1}\right)\times 100^2 \,. \quad (11S)$$

The standard deviation, which is taken as the noise of the cTnI/AT1 normalized responses is:

$$Stdev\left(\frac{cTnI}{AT1}\times 100\right) = \sqrt{Var\left(\frac{cTnI}{AT1}\times 100\right)} \,. \quad (12S)$$

**S3. Variance of normalized sandwich assay responses**

The normalized response for [(cTnI+AT2)/AT1] (g/g×100) is expressed as:

$$\frac{\Delta\Gamma(cTnI)+\Delta\Gamma(AT2)}{\Delta\Gamma(AT1)} = \left[\frac{\left(\frac{P_{out}(a_1)}{P_{out}(a_0)}-1\right)_{cTnI} + \left(\frac{P_{out}(a_1)}{P_{out}(a_0)}-1\right)_{AT2}}{\left(\frac{P_{out}(a_1)}{P_{out}(a_0)}-1\right)_{AT1}}\right]\times 100 \,. \quad (13S)$$

The available data in this case are:

| Means: | Variances: |
|---|---|
| $\mu_{cTnI(1)}$; $\mu_{cTnI(0)}$; | $V_{cTnI(1)}$; $V_{cTnI(0)}$; |
| $\mu_{AT2(1)}$; $\mu_{AT2(0)}$; | $V_{AT2(1)}$; $V_{AT2(0)}$; |
| $\mu_{AT1(1)}$; $\mu_{AT1(0)}$; | $V_{AT1(1)}$; $V_{AT1(0)}$; |

The variance for the AT2 response is (Eqs. (1S) and (2S)):

$$Var\left(AT2\right)=Var\left(\frac{P_{out}\left(a_1\right)}{P_{out}\left(a_0\right)}-1\right)_{AT2}=\frac{V_{AT2(1)}}{\left(\mu_{AT2(0)}\right)^2}+\frac{\left(\mu_{AT2(1)}\right)^2\times V_{AT2(0)}}{\left(\mu_{AT2(0)}\right)^4}. \quad (14S)$$

Considering equations (3S) and (5S), the variance and the mean of the numerator (cTnI+AT2) is given by:

$$Var\left(cTnI+AT2\right)=Var\left(cTnI\right)+Var\left(AT2\right). \quad (15S)$$

and

$$\mu\left(cTnI+AT2\right)=\frac{\mu_{cTnI(1)}}{\mu_{cTnI(0)}}+\frac{\mu_{AT2(1)}}{\mu_{AT2(0)}}-2. \quad (16S)$$

Thus, the total variance for the Sandwich Assay is expressed as (Eqs. (1S) and (2S)):

$$Var\left(\frac{cTnI+AT2}{AT1}\right)=\frac{Var\left(cTnI+AT2\right)}{\left(\mu_{AT1(1)}/\mu_{AT1(0)}\right)^2}+\frac{\left[\mu\left(cTnI+AT2\right)\right]^2\times Var\left(AT1\right)}{\left(\mu_{AT1(1)}/\mu_{AT1(0)}\right)^4}. \quad (17S)$$

where $Var\left(cTnI\right)$, $Var\left(AT\ \right)$, and $Var\left(AT2\right)$ are calculated using Eqs. (8S), (9S) and (14S) respectively. Incorporating a factor of 100, the total variance and standard deviation for the sandwich assay are given by:

$$Var\left(\frac{cTnI+AT2}{AT1}\times100\right)=Var\left(\frac{cTnI+AT2}{AT1}\right)\times100^2. \quad (18S)$$

and

$$Stdev\left(\frac{cTnI+AT2}{AT1}\times100\right)=\sqrt{Var\left(\frac{cTnI+AT2}{AT1}\times100\right)}. \quad (19S)$$

where $Stdev\left(\frac{cTnI+AT2}{AT1}\times100\right)$ was taken as the noise.
